# The Relationship Between Forgiveness and Health Outcomes Among People Living with HIV: A Cross-Sectional Study in France

**DOI:** 10.1007/s10461-023-04052-w

**Published:** 2023-04-24

**Authors:** Loren L. Toussaint, Sebastian Binyamin Skalski-Bednarz, Jean-Philippe Lanoix, Karol Konaszewski, Janusz Surzykiewicz

**Affiliations:** 1 Department of Psychology, Luther College, 700 College Dr, Decorah, IA 52101 USA; 2 Faculty of Education, Cardinal Stefan Wyszyński University in Warsaw, Warsaw, Poland; 3 Faculty of Philosophy and Education, Catholic University of Eichstätt-Ingolstadt, Eichstätt, Germany; 4 Department of Medicine, Johns Hopkins University School of Medicine, Baltimore, MD USA; 5 Faculty of Education, University of Białystok, Białystok, Poland

**Keywords:** Forgiveness, Self-forgiveness, Perceived stress, Health, Life satisfaction, PLWH, People living with HIV

## Abstract

Research to date has shown that HIV infection is a highly stressful experience for individuals, and one of the key adaptive resources after such painful experiences may be forgiveness. The aim of the present study was to examine the associations between dispositional forgiveness (assessed using Mullet’s Forgivingness Questionnaire and Toussaint’s Forgiveness Scale), perceived stress (single-item measure of stress symptoms), health perception (EuroQol visual analogue version of the scale) and life satisfaction (Satisfaction With Life Scale) in people living with HIV (PLWH) in France. Paper surveys were completed by 222 PLWH aged 18–78 (57% male). Multiple regression analysis revealed that sensitivity to circumstances, unconditional forgiveness, self-forgiveness, and forgiveness of others were significant predictors of health and happiness. Mediation analysis showed that these relationships are completely mediated by perceived stress. The present findings suggest that forgiveness and perceived stress may be important variables for healing in PLWH. Interventions designed to improve forgiveness and self-forgiveness may result in improved health and life satisfaction in PLWH.

## Introduction

According to the WHO [1], there are approximately 38 million people living with HIV (PLWH) worldwide. Although growing access to antiretroviral therapy (ART) has led to increased life expectancy in this population, a percentage of PLWH report co-occurring psychiatric disorders [2]. According to meta-analyses [3, 4], PLWH show an increased risk of post-traumatic stress disorder (PTSD). Being diagnosed with HIV as a life-threatening infection is in itself a potentially traumatic event because, if left untreated, it can cause progressive destruction of the immune system, which increases susceptibility to opportunistic infections and malignancies; furthermore, it is strongly associated with the occurrence of stigma [5]. On the other hand, Neigh and colleagues [6] indicate greater exposure to traumatic experiences by PLWH even before HIV diagnosis compared to the general population. That is, they are more likely to report childhood sexual/physical abuse or violence by close intimate partners. Other studies indicate higher prevalence of anxiety disorders and depressive symptoms [7] and personality disorders [8] and decreased life satisfaction [9, 10] among PLWH. Prevalence of psychiatric disorders in the seropositive population calls for research on psychosocial resources that may improve functioning in the face of experiencing a health crisis. HIV infection is a biopsychosocial phenomenon that affects not only physical health but also other aspects of PLWH’s lives, including occupational and social functioning, marriage and intimate relationships, and parenting [11]. Numerous conceptualizations suggest that biological, psychological, and behavioral factors interact in complex ways and influence the clinical progression of HIV-related diseases [12, 13]. At the same time, it is important to note that HIV is transmitted most often through two of the most intrinsic human forces – sexuality and procreation [14].

The fear and stigma surrounding HIV transmission in these most intimate human connections have given the multidimensional concept of forgiveness a central role for PLWH and their loved ones [15]. Forgiveness of others involves giving up the right to revenge and releasing the negative affect directed at the offender [16]. While a focus on forgiveness of others is common, researchers also consider multiple additional dimensions of the phenomenon [17, 18]. Self-forgiveness includes letting go of negative affect and self-blame for mistakes made [19]. Feeling forgiven by God refers to the belief that divinity forgives us for the wrongdoings we have committed [20]. One comprehensive and multifaceted approach to conceptualizing forgiveness was developed by Mullet and colleagues [21]. They developed a typology addressing virtually every possible circumstance of forgiveness: social proximity, severity of consequences, intent to harm, revenge, consequence cancellation, apology, pressure from loved ones and religious authorities, mood, as well as personal philosophy and faith. The authors identified three forgiveness dimensions cutting across these circumstances: (1) unconditional forgiveness is understood to reflect the general tendency of people to forgive (or seek revenge) regardless of motives; (2) sensitivity to circumstances assesses the influence of situational factors such as mood, reparations, apology, etc. on forgiveness; and (3) blockage to forgiveness explains a general disposition to not forgive regardless of motives or opportunities, and in further analyses has been associated with anxiety disorders and low self-esteem [21].

Temoshok and Wald [15] noted that the emotional and psychosocial consequences of forgiveness and feeling forgiven (or conversely unforgiveness/feeling unforgiveness) may have psychoneuroimmunological effects on the health of PLWH. The authors also indicated the occurrence of difficulties for PLWH in coming to terms with HIV (acceptance) and forgiving the person who they believe infected them. At the same time, Temoshok and Wald [15] pointed out the difficulty of self-forgiveness, especially when PLWH may have led to the infection of their partner or child, as well as when the infection occurred through male-male sexual contact or injection of drugs – both of the latter cases being associated with the so-called double social stigma [22]. Dispositional forgiveness in PLWH has also been shown to have important consequences for self-esteem, interpersonal relationships, and health and medical outcomes in the context of HIV/AIDS. Imasiku [23] noted that low levels of dispositional forgiveness can lead to increased psychosomatic complications as PLWH are unable to cope with stress and consequently cease to be immune to physical illness. Tiwari [22] indicated a positive relationship between self-forgiveness and life satisfaction in PLWH. Meanwhile, in a study by Martin and colleagues [24] PLWH reporting higher attachment anxiety and lower levels of forgiveness of others experienced greater suffering, while individuals reporting high levels of self-forgiveness presented a better perception of health. Thus, it appears that dispositional forgiveness may be one of the key adaptive resources after the painful experience of HIV infection.

Although the above literature review indicates that dispositional forgiveness may be beneficial to psychological functioning, we still do not clearly understand how forgiveness improves health perception and life satisfaction. According to the Stress-and-Coping Theories of Forgiveness of Others [25] and Self-Forgiveness [26], forgiveness can affect health through perceived stress. The development of these models has been based on Lazarus and Folkman’s [27] transactional theory of stress and coping, which posits that a stress response is a consequence of cognitive appraisal and occurs when a situation is judged to be taxing or beyond one’s available resources (coping abilities), thereby threatening one’s well-being. The model by Toussaint and colleagues [26] posits that (a) unforgiveness of others and self-unforgiveness, indexed by self- or other-directed anger, hatred, and resentment, creates stressful intrapersonal and interpersonal situations; (b) lack of forgiveness of others and self-forgiveness contributes in some part to the deleterious effects of stress on health; and (c) forgiveness of others and self-forgiveness are coping mechanisms that are able to reduce stress experiences associated with unforgiveness of others or self-unforgiveness. Toussaint and colleagues [26, 28] indicate that forgiveness of others and self-forgiveness are not the only coping strategies available, but according to their model, they are two of the more effective responses for reducing stress and improving health. Thus, it appears that the relationship of forgiveness of others and self-forgiveness with perceptions of health and life satisfaction may be mediated by levels of perceived stress.

The purpose of this study was to further our understanding of the relationship of multiple dimensions of forgiveness with quality of life in PLWH. We tested the following two hypotheses: (a) dimensions of forgiveness would be positive predictors of health perception and life satisfaction in PLWH and (b) positive associations between dimensions of forgiveness and health perception and life satisfaction in PLWH would be mediated by lower levels of perceived stress.

## Methods

### Participants and Procedure

Anonymous data from a paper-and-pencil survey were collected between 2020 and 2021. Because of the design of the study, approval of the university ethics committee was waived by French law. PLWH were recruited from among patients at the University Hospital of Amiens in northern France (a diagnosis of HIV infection was the only inclusion criteria). By international recommendations, all subjects received ART medication. The study procedure consisted of completing questionnaires to assess forgiveness, perceived stress, health perception, and life satisfaction. Participation was anonymous and voluntary, preceded by oral informed consent. In addition, each participant was informed of the objectives and procedure. The final cohort consisted of 222 PLWH (57% male) aged 18–78 *(M* = 48.62, *SD =* 12.56). Most participants (53%) were in a relationship. Of those in relationships, all women reported being in female-male relationships, while 47% of men reported being in male-male relationships.

### Measures

The *Forgivingness Questionnaire (FQ)* by Mullet and colleagues [21] was used to assess the circumstances of forgiveness. This French scale consists of 15 statements arranged into three factors: blockage to forgiveness *(α* = 0.78), sensitivity to circumstances *(α* = 0.75), and unconditional forgiveness. (*α* = 0.82). An example item from the blockage to forgiveness subscale is, “As far as I am concerned, I don’t feel able to forgive even if the offender has apologized.” An example item from the sensitivity to circumstances subscale is “As far as I am concerned, I forgive more easily when I feel good and everything goes well.” An example item from the unconditional forgiveness subscale is “As far as I am concerned, I can easily forgive even if the consequences of the harm done are serious ones.” Respondents rate each of the statements on a 11-point Likert scale of 1 (*I strongly disagree*) to 11 (*I strongly agree*).

**Fig. 1:**
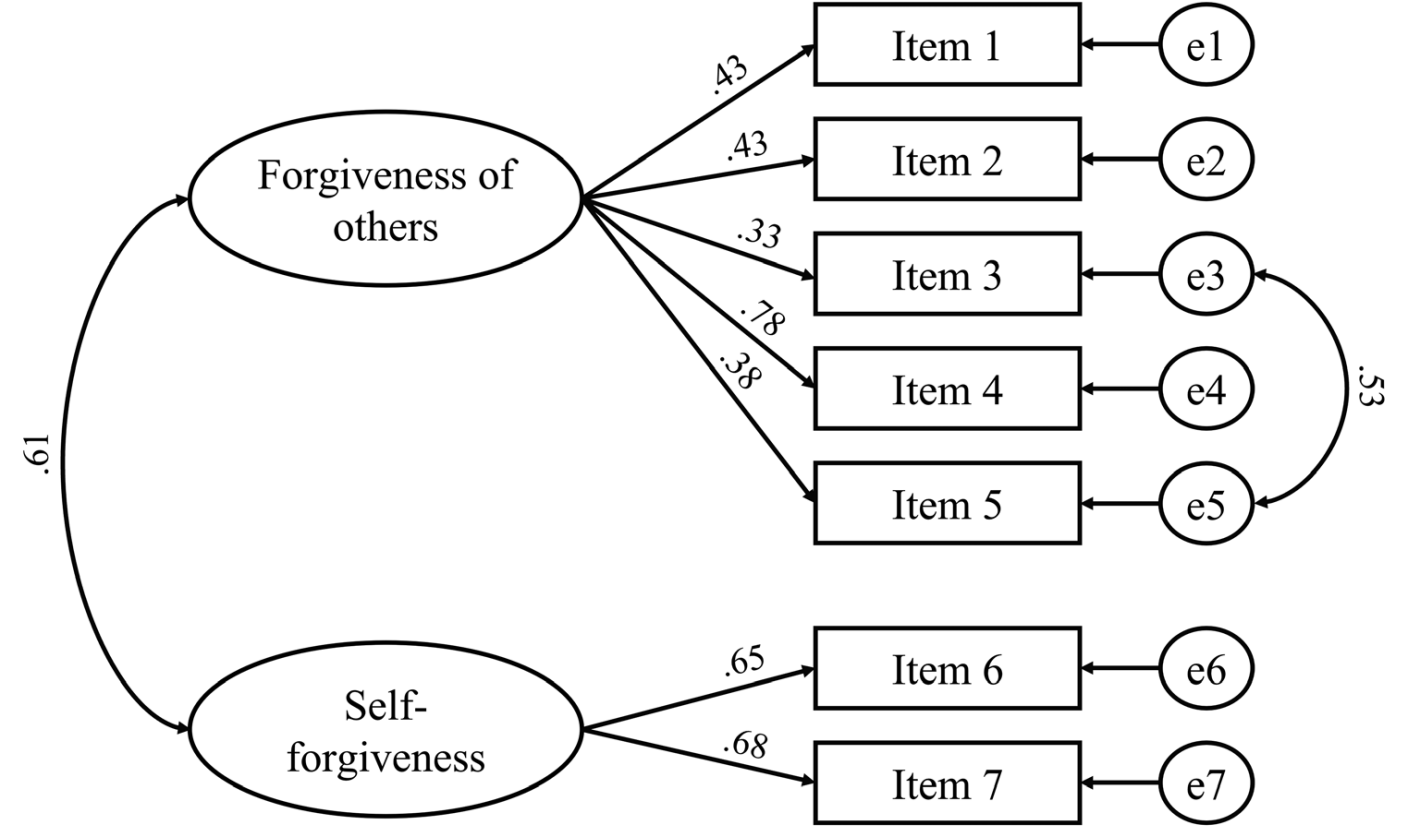
Two-factor structure of the French version of the Forgiveness Scale (N = 222)

The *Forgiveness Scale (FS)* by Toussaint and colleagues [29] was used to assess forgiveness of others and self-forgiveness dispositions. An example item from the forgiveness of others scale is, “I have forgiven those who have hurt me.” An example item from the self-forgiveness subscale is, “I find it hard to forgive myself for some of the things I have done wrong.” The original version of FS was translated into French by three independent translators with a high proficiency in English. The translations were adjusted to the final version of the scale by the authors of the present study. Next, the final version was back-translated into English by two independent translators with a high level of proficiency in English. Any differences between the original and back-translated version of the scale were discussed and amended by four authors of the study and the final version of the FS was accepted by the author of the scale. The translation of the scale was carried out in accordance with accepted principles developed for the purposes of intercultural research, based on the original English version. Respondents rated each of the statements on a five-point Likert scale of 1 (*I strongly disagree*) to 5 (*I strongly agree*). As with the original version of the FS, confirmatory factor analysis (CFA) revealed a two factor structure (see Fig. 1) for seven statements in the French version of the scale. Estimates of internal consistency for the French version were acceptable for short scales: forgiveness of others (*α* = 0.65) and self-forgiveness (*α* = 0.57) [30, 31]. The model proved to be a good fit to the data: *χ*^2^[12] = 11.87; p = .456 ; *χ*^2^/df = 0.99; RMSEA = 0.002 (0.001,0.038;90% CI); SRMR = 0.02; CFI = 0.99.

The single-item measure of stress symptoms [32–34] was used to assess stress, and the same procedures used to translate the forgiveness items were used to translate the stress item. The question was: *“Stress means a situation when a person feels tense, restless, nervous, or anxious, or is unable to sleep at night because his or her mind is troubled all the time. Do you feel that kind of stress these days?”* The response was made on a five-point Likert scale varying from 1 (*not at all*) to 5 (*very much*).

To assess health perception, we used the *EuroQol (EQ)* version of the *visual analogue scale (VAS)* developed by the EuroQol Group [35]. The EQ VAS records the participant’s self-rated health in response to the question, “*We would like to know how good or bad your health is today.*” Responses were made on a vertical visual analogue scale, where the endpoints are labeled 100 (*The best health you can imagine*) and 0 (*The worst health you can imagine*). The VAS is used as a quantitative measure of health that reflects the patient’s own judgment.

To assess life satisfaction, we used the Satisfaction With Life Scale (SWLS) developed by Diener and colleagues [36] in French [37]. The SWLS consists of five statements arranged in one factor (*α* = 0.80). An example item is, “I am satisfied with my life.” Respondents rate each of the statements on a five-point Likert scale of 1 (*I strongly disagree*) to 5 (*I strongly agree*).

In addition, participants completed demographic questionnaires and were asked to identify their religiosity and spirituality on a four-point Likert scale of 1 *I am not*) to 4 (*I am very*) (in both cases).

### Statistical Analyses

We used hot-deck imputation to address missing data. Hot-deck imputation is suitable for situations where there is less than 20% missingness [38]. This method of imputation replaces missing data for a given respondent with complete data from a matched respondent selected randomly from a group of matched respondents. Data were 9, 13, and 7% missing for health perceptions, sex, and self-forgiveness, respectively. All other variables had 5% or less missing data. The Kolmogorov-Smirnov test was used to assess normal distribution. Levene’s test was used to assess homoscedasticity. The scores obtained allowed for application of parametric tests. Confirmatory factor analysis (CFA) with maximum likelihood (ML) estimation implemented was applied to assess the factor structure of FS (see “Measures”). The chi-square statistic (*χ*^*2*^) was used to assess the sample and the implied covariance matrices (an acceptable value of *χ*^*2*^/df is less than 2); however, this statistic strongly depends on sample size and provides an overly conservative assessment of model fit. Therefore, additional recommended measures of fit were also examined [39, 40]. The comparative fit index (CFI) was used to assess model fit relative to a baseline model in which all variables are uncorrelated, and values above 0.95 indicate good fit, while values above 0.90 are considered to indicate acceptable fit. The root-mean-square error of approximation (RMSEA) was also provided. Ideally, this should be less than 0.05, but values less than 0.08 are considered to be acceptable. Moreover, the standardized root mean square residual (SRMR) was included, which should be less than 0.08 for proper fit. Pearson’s correlation analysis and regression analysis were used to determine the relations between the variables. The mediation model was assessed using Hayes’ PROCESS macro (model no. 4). The significance level was determined at *p* < .05. The effect size was assessed based on *R*^*2*^. Data analysis was conducted in IBM SPSS Statistics 27 and IBM SPSS Amos 27.

## Results

### Correlation Analysis

In the first step, we conducted a correlation analysis (see Table 1). In terms of our main variables, we observed statistically significant associations between:

**Table 1:** Means and correlations (N = 222)

	M (SD)	1.	2.	3.	4.	5.	6.	7.	8.
1. Blockage to forgiveness	4.42 (2.71)	-							
2. Sensitivity to circumstances	5.53 (2.86)	0.43***	-						
3. Unconditional forgiveness	5.42 (2.89)	−0.02	0.13	-					
4. Forgiveness of others	3.36 (0.89)	−0.41***	−0.24***	0.33***	-				
5. Self-forgiveness	2.95 (1.22)	−0.21**	−0.22***	0.05	0.32***	-			
6. Perceived stress	2.36 (1.11)	0.10	0.08	−0.15*	−0.37***	−0.23***	-		
7. Perception of health	74.35 (19.26)	0.05	0.17**	0.19**	0.14*	0.22***	−0.40***	-	
8. Satisfaction with life	4.54 (1.62)	−0.02	−0.01	0.11	0.28***	0.17**	−0.49***	0.45***	-
Sex (0 = male, 1 = female)		0.06	−0.06	−0.04	0.01	−0.07	−0.05	0.07	−0.07
Age	48.36 (12.41)	0.01	0.02	−0.05	−0.01	−0.06	0.09	−0.08	0.04
Relationship (0 = no, 1 = yes)		−0.05	−0.04	−0.01	0.02	0.03	−0.17**	0.14*	0.27***
In relationship with… (0 = male, 1 = female)		−0.13	0.01	0.06	0.12	−0.11	0.01	−0.09	−0.12
Sex * In relationship with…		0.30***	0.23***	0.13	0.10	−0.26***	−0.08	−0.04	−0.08
Religiosity	2.21 (1.14)	−0.02	−0.01	0.19**	0.11	0.01	−0.11	−0.05	0.01
Spirituality	2.40 (1.17)	−0.14*	−0.11	0.15*	0.15*	0.04	0.02	−0.04	0.01

* p < .05; ** p < .01; *** p < .001

- More blockage to forgiveness and more sensitivity to circumstances, less forgiveness of others, and less self-forgiveness;
- More sensitivity to circumstances, more forgiveness of others, more self-forgiveness, and better perception of health;
- More unconditional forgiveness and more forgiveness of others, less perceived stress, and better perception of health;
- More forgiveness of others and more self-forgiveness, less perceived stress, better perception of health, and more satisfaction with life;
- More self-forgiveness and less perceived stress, better perception of health, and more satisfaction with life;
- More perceived stress and worse perception of health and more satisfaction with life;
- Better perception of health and more satisfaction with life.

In addition, being in a relationship was associated with less perceived stress, better perception of health, and more satisfaction with life. Religiosity correlated with more unconditional forgiveness, while spirituality correlated with less blockage to forgiveness, more unconditional forgiveness, and more forgiveness of others. We also observed that participants in male-male relationships reported more blockage to forgiveness, more sensitivity to circumstances, and less self-forgiveness compared to men and women in heterosexual relationships (women in same-sex relationships did not participate in this study). Other relationships proved to be statistically nonsignificant.

### Regression Analysis

We then conducted regression analyses where health perception and life satisfaction served as dependent variables, while forgiveness factors served as independent variables (see Table 2). In the first step of the regression model, predictors included blockage to forgiveness, sensitivity to circumstances, and unconditional forgiveness. In the second step, forgiveness of others and self-forgiveness were added. All five dimensions were included in the third step of the model. This model proved to be the best fit to the data and was able to account for 15% of the variance in perception of health and 11% of the variance in satisfaction with life. The analyses showed that unconditional forgiveness, self-forgiveness, and sensitivity to circumstances (but this one only in the third model) were significant predictors of perception of health, while forgiveness of others helped predict satisfaction with life. Detailed values of regression coefficients are presented in Table 2. Before performing the regression analysis, we performed a collinearity test (Variance Inflation Factor; VIF) due to the presence of correlations between predictors. The variance inflation factor (VIF parameter) did not exceed the maximum value of 10 [41]. Hence, the independent variables were not collinear.

**Table 2:** Forgiveness factors as predictors of health perceptions and life satisfaction (N = 222)

	Perception of health^a^			Satisfaction with life^b^		
Variable	**B**	**SE**	**β**	**B**	**SE**	**β**
**Model 1**						
Blockage to Forgiveness	0.13	0.63	0.02	0.01	0.05	0.02
Sensitivity to circumstances	1.00	0.58	0.15	−0.01	0.05	−0.02
Unconditional Forgiveness	1.34	0.53	0.20***	0.07	0.04	0.13
**Model 2**						
Forgiveness of others	2.77	2.12	0.11	0.51	0.15	0.25***
Self-forgiveness	2.95	1.38	0.18**	0.12	0.10	0.09
**Model 3**						
Blockage to Forgiveness	0.17	0.69	0.02	0.07	0.05	0.11
Sensitivity to circumstances	1.41	0.62	0.20*	0.03	0.05	0.06
Unconditional forgiveness	1.28	0.61	0.18*	0.00	0.04	0.01
Forgiveness of others	1.94	2.46	0.08	0.57	0.18	0.28***
Self-forgiveness	3.75	1.34	0.23**	0.17	0.10	0.13

* p < .05; ** p < .01; *** p < .001;

^a^ Model 1: *F*_(3,219)_ = 3.16, *p* = .026, *R*^*2*^ = 0.08; Model 2: *F*_(2,220)_ = 4.62, *p* = .011, *R*^*2*^ = 0.07; Model 3: *F*_(5,217)_ = 4.84, *p* < .001, *R*^*2*^ = 0.15

^b^ Model 1: *F*_(3,219)_ = 1.01, *p* = .389, *R*^*2*^ = 0.03; Model 2: *F*_(2,220)_ = 8.49, *p* < .001, *R*^*2*^ = 0.09; Model 3: *F*_(5,217)_ = 4.01, *p* = .002, *R*^*2*^ = 0.11

### Mediation Analysis

Mediation analyses were then performed using bootstrap sampling (5000) with 95% confidence intervals. In all models, perceived stress was evaluated as a potential mediator between forgiveness factors and perception of health or satisfaction with life (see Table 3). A significant mediation effect of perceived stress was obtained in the relationship between unconditional forgiveness and perception of health. The overall effect (c path) equaled *B* = 1.39 (*t* = 2.62, *p* = .005; *R*^*2*^ = 0.04). The regression coefficient of the independent variable’s impact on the mediator (a path) was *B* = − 0.06 (*t* = − 1.77, *p* = .039; *R*^*2*^ = 0.03), the regression coefficient of the mediator’s impact on the dependent variable while controlling the independent variable (b path) was *B* = − 6.90 (*t* = − 5.01, *p* < .001; *R*^*2*^ for the whole model = 0.17). Stress decreased the strength of the association between unconditional forgiveness and perception of health to insignificant; the direct effect (c′ path) was *B* = 0.89 (*t* = 1.61, *p* = .066). In the model assessing the association between forgiveness of others and perception of health, c path was *B* = 2.94 (*t* = 1.69, *p* = .046; *R*^*2*^ = 0.02), a path equaled *B* = − 0.49 (*t* = − 5.54, *p* < .001; *R*^*2*^ for the entire model = 0.14), b path equaled *B* = − 7.60 (*t* = − 5.16, *p* < .001; *R*^*2*^ for the entire model = 0.16), and c′ path was B = − 0.68 (*t* = − 0.37, *p* = .357). The c path for self-forgiveness and perception of health totaled *B* = 3.37 (*t* = 3.56, *p* < .001; *R*^*2*^ = 0.07), a path totaled *B* = − 0.21 (*t* = − 3.23, *p* < 001; *R*^*2*^ = 0.05), b path totaled *B* = − 7.88 (*t* = − 5.27, *p* < .001; *R*^*2*^ for the entire model = 0.19) with the c′ path totaling *B* = 1.12 (*t* = 0.87, p = .193).

**Table 3:** The mediating role of stress perception in relationships between forgiveness factors and health outcomes (N = 222)

	a Path			b Path			c Path			c′ Path			Indirect Effect and B (SE)	95% CI LOWER UPPER
	B	SE	β	B	SE	β	B	SE	β	B	SE	β		
Outcome: perception of health														
BF → S → PH	0.02	0.04	0.11	−7.58	1.34	−0.41***	0.37	0.58	0.05	0.63	0.54	0.08	−0.261 (0.247)	−0.763; 0.223
SC → S → PH	0.03	0.03	0.08	−7.60	1.32	−0.41***	1.23	0.53	0.17**	1.37	0.49	0.20*	−0.137 (0.230)	−0.615; 0.315
UF → S → PH	−0.06	0.03	−.015*	−6.90	1.38	−0.37***	1.39	0.52	0.19**	0.89	0.51	0.12	0.501 (0.253)	0.063; 1.043
FO → S → PH	−0.49	0.09	−0.37***	−7.60	1.47	−0.41***	2.94	1.86	0.14*	−0.68	1.86	−0.03	3.620 (1.057)	1.792; 5.928
SF → S → PH	−0.21	0.06	−0.23***	−7.88	1.50	−0.41***	3.37	1.33	0.22***	1.12	1.29	0.07	2.245 (0.694)	1.045; 3.733
Outcome: satisfaction with life														
BF → S → SL	0.02	0.04	0.11	−0.74	0.09	−0.50***	−0.01	0.04	−0.02	0.02	0.04	0.03	−0.033 (0.023)	−0.079; 0.011
SC → S → SL	0.03	0.03	0.08	−0.75	0.09	−0.51***	−0.01	0.04	−0.01	0.02	0.03	0.04	−0.023 (0.021)	−0.064; 0.018
UF → S → SL	−0.06	0.03	−.015*	−0.75	0.09	−0.51***	0.06	0.04	0.11	0.02	0.03	0.04	0.042 (0.022)	−0.001; 0.089
FO → S → SL	−0.49	0.09	−0.37***	−0.68	0.10	−0.46***	0.57	0.14	0.28***	0.23	0.13	0.12	0.332 (0.077)	0.189; 0.495
SF → S → SL	−0.21	0.06	−0.23***	−0.79	0.09	−0.53***	0.23	0.10	0.17**	0.07	0.09	0.05	0.163 (0.055)	0.065; 0.276

* p < .05; ** p < .01; *** p < .001; BF = blockage to forgiveness, SC = sensitivity to circumstances, UF = unconditional forgiveness, FO = forgiveness of others, SF = self-forgiveness, S = perceived stress, PH = perception of health, SL = satisfaction with life; a path = effect of the independent variable on the mediator; b path = effect of the mediator on the dependent variable; c path = effect of the independent variable on the dependent variable; c′ path = direct effect of the independent variable on the dependent variable while controlling for the mediator. Effects are adjusted for marital status (i.e., being in a relationship)

The c path for forgiveness of others and satisfaction with life totaled *B* = 0.57 (*t* = 4.19, *p* < .001; *R*^*2*^ = 0.08), b path totaled *B* = − 0.68 (*t* = − 6.99, *p* < .001; *R*^*2*^ for the entire model = 0.26) with the c′ path totaling *B* = 0.23 (*t* = 1.59, p = .056). The c path for self-forgiveness and satisfaction with life totaled *B* = 0.23 (*t* = 2.36, *p* = .009; *R*^*2*^ = 0.03), b path totaled *B* = − 0.79 (*t* = − 8.33, *p* < .001; *R*^*2*^ for the entire model = 0.29) with the c′ path totaling *B* = 0.07 (*t* = 0.77, p = .221). The other relationships between forgiveness factors and perception of health or satisfaction with life were not mediated by perceived stress. The unstandardized and standardized coefficients and their 95% confidence intervals are provided in Table 3.

## Discussion

According to previous research, the experience of HIV infection is a highly stressful event for individuals and is associated with co-occurring mental disorders [8, 11]. The purpose of the present study was to assess the dimensions of forgiveness as predictors of quality of life in PLWH. The analyses conducted showed that sensitivity to circumstances, unconditional forgiveness and self-forgiveness were significant predictors of health perception, which corresponds with previous findings [15, 22]. Nkomo and Kufankomwe [42] noted that self-forgiveness is essential for PLWH to assume the patient’s social role, undergo ART treatment, and adhere to treatment. Hua [43] indicated that shame prevents PLWH from seeking and accessing medical care and disclosing their HIV status. In our study, forgiveness of others was found to be more important for the development of life satisfaction in PLWH compared to self-forgiveness, which appears to be contrary to research on healthy individuals [44, 45]. A possible explanation for this effect is the finding of Mauger and colleagues [46], according to which unforgiveness of others develops the desire for revenge and interpersonal alienation, whereas the critical role of social exclusion and alienation has been widely demonstrated in predicting life satisfaction in PLWH [47, 48]. Another explanation would be that France, being a very secular country where forgiveness is not taught or preached widely, might be a place where people who forgive others would feel a greater satisfaction in doing it than would people in other countries. Another finding of interest is that the sensitivity to circumstances factor proved to be a non-significant predictor of health perception in a model considering only the factors proposed in the conceptualization by Mullet and colleagues [21]. Only the inclusion of recipients of forgiveness dispositions (forgiveness of others and self-forgiveness) increased the predictive value of sensitivity to circumstances to statistically significant and further improved the validity of the entire model. In other words, the sensitivity to circumstances measure introduced an external measurement error variance for the results of the forgiveness of others and self-forgiveness measures, that is, a measurement artifact variance. This observation suggests that although self-forgiveness is an important variable for securing quality of life in PLWH, noticing stimuli from the social environment and the appropriate emotional valence of these stimuli (i.e., sensitivity to circumstances) are necessary for self-forgiveness to occur for things PLWH have done wrong.

The conducted mediation analyses deepened the understanding of how forgiveness improves perception of health and life satisfaction. According to the data obtained, PLWH who forgive themselves and others show lower levels of stress, which contributes to better perceived health and greater life satisfaction (for perceived health, the significant mediation effect was also related to the unconditional forgiveness index). The data obtained provide empirical support for the assumptions of the stress and coping theories of forgiveness and self-forgiveness [25, 26]. The results are consistent also with prior research showing the association of forgiveness with health may be explained by stress [28]. Similarly, Griffin and colleagues [49] noted that unforgiveness is a stress response associated with poor mental health, while various psychological states may mediate the effects of forgiveness on health.

In our study, PLWH who were in relationships reported lower levels of stress and better perception of health and life satisfaction than those who were single. This seems understandable, as research to date indicates that social support is a key environmental variable that promotes adaptation and healing processes [50]. Meanwhile, participants in male-male relationships exhibited higher scores on blockage to forgiveness, sensitivity to circumstances, and lower self-forgiveness (women in same-sex relationships did not participate in this study). This observation, however, does not appear to be directly related to marital status, but rather to participants’ sexual orientation (we did not explicitly ask about orientation in the survey). Indeed, people with same-sex attractions with HIV experience the phenomenon of double social stigma – both because of their infection and their different orientation [22]. It is also important to note that religiosity was associated with more unconditional forgiveness and spirituality further was associated with more forgiveness of others and limited blockage to forgiveness, a common observation in the literature [21, 29, 51]. Some data even suggest that forgiveness is an important pathway through which the effects of religion-related variables have their effect on health [52]. In our study, however, we did not conduct similar analyses due to the high secularization rate of French society [53], which would likely distort the results obtained.

The data obtained make an important contribution to existing knowledge regarding the role of forgiveness in health in PLWH. Before generalizing more broadly, however, some limitations of this study must be considered. First of all, data were collected from a small sample from France. Further research among other populations is needed to generalize conclusions. Secondly, all individuals were treated with ART. This means that they accepted HIV infection, accepted the social role of the patient, and made the effort to safeguard their health. Data from untreated PLWH may differ significantly from the results obtained in this study. Finally, the study did not control for comorbidities of other chronic diseases and participants’ actual health status, such as viral load levels and CD4 cell counts were not measured (only subjective perceptions of health were assessed). Consideration of these data in future research (e.g., assessing the association of forgiveness and objective measures of health) may significantly increase the utility of the findings. In future work, it also seems worthwhile to examine intervention techniques and employ longitudinal studies capable of ascertaining causal effects.

The present study is one of the few studies that evaluates the relationship of multiple dimensions of forgiveness and health in PLWH. The data obtained showed that dispositional forgiveness (both of self and others) may be associated with better health outcomes in PLWH. Our data also allow us to extend previous findings on the effects underlying the interplay between dimensions of forgiveness and health. The present findings are the first to show that the associations of forgiveness and perception of health and life satisfaction in PLWH can be explained by reductions in levels of perceived stress. Future studies examining the stress-and-coping theories of forgiveness and self-forgiveness in PLWH will allow us to continue to better understand these associations, evaluate the effects of forgiveness and self-forgiveness interventions, and provide improved evidence to support their use in the psychosocial care of PLWH.

The present findings, while part of a small literature, do suggest some important areas of application in the clinical care of people living with HIV. First, forgiveness issues may require careful screening and/or assessment in the clinical setting. If forgiveness issues are related to health outcomes as the present study indicates, then identifying and addressing these issues might need to be included in the care of people living with HIV. This may require focused attention on the development and testing of both screening tools and more sophisticated assessments. Second, it may be necessary to provide training to ready healthcare professionals for these important discussions and to make them aware of the implications of forgiveness struggles for patient’s health and well-being. Third, if forgiveness issues are identified, providers should have resources available to refer patients to. In this regard, many resources can be found on the internet (e.g., www.forgivenessfoundation.org), and several books, workbooks, and audio/video resources are available. Finally, healthcare providers should take the findings from the present study and use them to continue to advocate against stigmatization of people living with HIV by leveraging both forgiveness and self-forgiveness to promote inclusivity and equitable treatment of people living with HIV not only in healthcare settings but in community and cultural contexts. With continued growth in attention to this important concern for people living with HIV, healthcare professionals can continue to learn and support the health and well-being of their patients and promote the healthy resolution of forgiveness issues that provide improved quality of life.

## Data Availability

The datasets generated during and/or analyzed during the current study are available from the corresponding author on reasonable request.
