# Correction to: The Relationship Between Forgiveness and Health Outcomes Among People Living with HIV: A Cross-Sectional Study in France

**DOI:** 10.1007/s10461-023-04101-4

**Published:** 2023-06-17

**Authors:** Loren L. Toussaint, Sebastian Binyamin Skalski-Bednarz, Jean-Philippe Lanoix, Karol Konaszewski, Janusz Surzykiewicz

**Affiliations:** 1 Department of Psychology, Luther College, 700 College Dr., Decorah, IA 52101 USA; 2 Faculty of Education, Cardinal Stefan Wyszyński University in Warsaw, Warsaw, Poland; 3 Faculty of Philosophy and Education, Catholic University of Eichstätt-Ingolstadt, Eichstätt, Germany; 4 Department of Medicine, Johns Hopkins University School of Medicine, Baltimore, MD USA; 5 Faculty of Education, University of Białystok, Białystok, Poland

**Correction to: AIDS and Behavior** **https://doi.org/10.1007/s10461-023-04052-w**

Due to a mistake on the Publisher’s end, the original version of this article contained errors in Table 3. Table 3 has been corrected in this version of the article. Springer Nature apologizes for the errors.

The original article has been corrected.

**Table 3:** The mediating role of stress perception in relationships between forgiveness factors and health outcomes (N = 222)

	a Path			b Path			c Path			c′ Path			Indirect effect and B (SE)	95% CI Lower; Upper
	B	SE	β	B	SE	β	B	SE	β	B	SE	β		
*Outcome: perception of health*														
BF → S → PH	0.02	0.04	0.11	− 7.58	1.34	− 0.41***	0.37	0.58	0.05	0.63	0.54	0.08	− 0.261 (0.247)	− 0.763; 0.223
SC → S → PH	0.03	0.03	0.08	− 7.60	1.32	− 0.41***	1.23	0.53	0.17**	1.37	0.49	0.20*	− 0.137 (0.230)	− 0.615; 0.315
UF → S → PH	− 0.06	0.03	− .015*	− 6.90	1.38	− 0.37***	1.39	0.52	0.19**	0.89	0.51	0.12	0.501 (0.253)	0.063; 1.043
FO → S → PH	− 0.49	0.09	− 0.37***	− 7.60	1.47	− 0.41***	2.94	1.86	0.14*	− 0.68	1.86	− 0.03	3.620 (1.057)	1.792; 5.928
SF → S → PH	− 0.21	0.06	− 0.23***	− 7.88	1.50	− 0.41***	3.37	1.33	0.22***	1.12	1.29	0.07	2.245 (0.694)	1.045; 3.733
*Outcome: satisfaction with life*														
BF → S → SL	0.02	0.04	0.11	− 0.74	0.09	− 0.50***	− 0.01	0.04	− 0.02	0.02	0.04	0.03	− 0.033 (0.023)	− 0.079; 0.011
SC → S → SL	0.03	0.03	0.08	− 0.75	0.09	− 0.51***	− 0.01	0.04	− 0.01	0.02	0.03	0.04	− 0.023 (0.021)	− 0.064; 0.018
UF → S → SL	− 0.06	0.03	− .015*	− 0.75	0.09	− 0.51***	0.06	0.04	0.11	0.02	0.03	0.04	0.042 (0.022)	− 0.001; 0.089
FO → S → SL	− 0.49	0.09	− 0.37***	− 0.68	0.10	− 0.46***	0.57	0.14	0.28***	0.23	0.13	0.12	0.332 (0.077)	0.189; 0.495
SF → S → SL	− 0.21	0.06	− 0.23***	− 0.79	0.09	− 0.53***	0.23	0.10	0.17**	0.07	0.09	0.05	0.163 (0.055)	0.065; 0.276

*p < 0.05; **p < 0.01; *** p < 0.001. BF = blockage to forgiveness, SC = sensitivity to circumstances, UF = unconditional forgiveness, FO = forgiveness of others, SF = self-forgiveness, S = perceived stress, PH = perception of health, SL = satisfaction with life; a path = effect of the independent variable on the mediator; b path = effect of the mediator on the dependent variable; c path = effect of the independent variable on the dependent variable; c′ path = direct effect of the independent variable on the dependent variable while controlling for the mediator. Effects are adjusted for marital status (i.e., being in a relationship)

